# Supplementary material for: Harnessing ultraconfined graphene plasmons to probe the electrodynamics of superconductors
Source: Proc Natl Acad Sci U S A. 2021 Jan 21;118(4):e2012847118. doi: 10.1073/pnas.2012847118 (PMC7848587; doi:10.1073/pnas.2012847118)
Supplement: Supplementary File [file pnas.2012847118.sapp.pdf]

PNAS
www.pnas.org

Supplementary Information for

Harnessing ultraconfined graphene plasmons to probe the electrodynamics of superconductors

A. T. Costa, P. A. D. Gonçalves, D. N. Basov, Frank H. L. Koppens, N. Asger Mortensen, N. M. R. Peres

Corresponding author: N. M. R. Peres, N. Asger Mortensen.
E-mail: peres@fisica.uminho.pt, asger@mailaps.org

This PDF file includes:

Supplementary text
Figs. S1 to S3
SI References

Supporting Information Text

S1. Details on the Semiclassical Infinite Barrier (SCIB) Model

S1.1. Nonlocal conductivity of the superconductor. In general, the full nonlocal conductivity tensor of a Cooper-paired superconductor is burdensome to deal with due to its intricated dependence on both wave vector $\mathbf{q}$ and frequency ω (1). Nonetheless, since we are interested in investigating surface electromagnetic waves with in-plane (i.e., parallel to the superconductor's surface) wave vectors much smaller that the superconductor's electronic Fermi wave vector k_F, it is sufficient to treat nonlocality to leading-order in q. In this limit, the superconductor's nonlocal conductivity tensor takes the form (1–3)

$$\overleftrightarrow{\boldsymbol{\sigma}}(\mathbf{q},\omega) = \sigma_\mathrm{D}(\omega)\left[\overleftrightarrow{\mathbf{I}} + \bar{\alpha}(\omega,T)\left(\frac{qc}{\omega}\right)^2\left(\overleftrightarrow{\mathbf{I}} + 2\hat{\mathbf{q}}\otimes\hat{\mathbf{q}}\right)\right], \tag{1a}$$

where $\sigma_\mathrm{D}(\omega) = \dfrac{ine^2}{m(\omega + \mathrm{i}\gamma)}$ is the local, Drude-type conductivity and $\hat{\mathbf{q}} \equiv \dfrac{\mathbf{q}}{|\mathbf{q}|}$ is a unit vector along the direction of $\mathbf{q}$. The dimensionless function $\bar{\alpha}(\omega,T)$ is given by (1–3)

$$\bar{\alpha}(\omega,T) = \frac{\hbar^4}{30\pi^2 n m^3 c^2}\int_0^\infty dk\, k^6 \left\{ \frac{2f(E_\mathbf{k})[1-f(E_\mathbf{k})]}{k_\mathrm{B}T}\left[1 - \frac{\Delta_0^2(T)}{E_\mathbf{k}^2}\right] + \frac{(\hbar\omega)^2\Delta_0^2(T)}{E_\mathbf{k}^3}\frac{1-2f(E_\mathbf{k})}{(\hbar\omega)^2 - (2E_\mathbf{k})^2}\right\}, \tag{1b}$$

and accounts for electronic processes governing the superconductor's response; specifically, the first and second terms of Eq. (1b) describe, respectively, quasiparticle scattering and two-quasiparticle absorption and emission processes. Here, we have

$$E_\mathbf{k} = \sqrt{(\varepsilon_\mathbf{k} - \mu)^2 + \Delta_0^2(T)} \qquad \left[\mu \simeq E_\mathrm{F} = \frac{\hbar^2}{2m}(3\pi^2 n)^{2/3} \quad \text{and} \quad \varepsilon_\mathbf{k} = \frac{\hbar^2 k^2}{2m}\right],$$

$$\Delta_0(T) = 1.76 k_\mathrm{B} T_c [1 - (T/T_c)^4]^{1/2}\Theta(T_c - T),$$

$$f(E_\mathbf{k}) = [\exp(E_\mathbf{k}/k_\mathrm{B}T) + 1]^{-1},$$

where we have assumed that the superconductor's gap parameter is energy-independent, i.e., $\Delta_0(T) \approx \Delta_\mathbf{k}(T)$.

The nonlocal conductivity tensor $\overleftrightarrow{\boldsymbol{\sigma}}(\mathbf{q},\omega)$ can be made diagonal by writing it in terms of longitudinal and transverse components, reading (2, 3)

$$\sigma_\mathrm{L}(q,\omega) = \sigma_\mathrm{D}(\omega)\left[1 - 3\bar{\alpha}(\omega,T)\left(\frac{qc}{\omega}\right)^2\right]^{-1}, \tag{2}$$

$$\sigma_\mathrm{T}(q,\omega) = \sigma_\mathrm{D}(\omega)\left[1 + \bar{\alpha}(\omega,T)\left(\frac{qc}{\omega}\right)^2\right], \tag{3}$$

respectively. Here, the electrodynamic response of the superconductor was incorporated through a Taylor expansion—up to second order in q/k_F, where k_F is the Fermi wave vector of the superconductor in the normal state—of the RPA-like nonlocal (i.e., q-dependent) conductivity tensor of the superconductor. This is justified because the limit $q \ll k_\mathrm{F}$ is well-realized here (for instance, throughout this work, k_F is at least two orders or magnitude larger than the in-plane wave vectors $q_\parallel$ associated with the polariton modes investigated here). Further, the limit $q\xi_\mathrm{BCS} \ll 1$, where $\xi_\mathrm{BCS} = \hbar v_\mathrm{F}/(\pi\Delta_0)$ denotes the superconductor's coherence length, is also satisfied which legitimates a mean-field-type treatment like the one adopted here.

S1.2. Semiclassical infinite barrier model. Following Rickayzen and Keller (1–3), we employ the semiclassical infinite barrier (SCIB) model (4, 5) to describe surface electromagnetic waves supported by a planar dielectric–superconductor interface. In particular, we seek the reflection coefficient for p-polarized waves associated with a planar dielectric–superconductor interface, since the system's collective excitations can be identified by analyzing its poles (which, in turn, manifest themselves as peaks in $\operatorname{Im} r_p$). In what follows, we present a concise description of the main steps leading to the derivation* of the reflection coefficient for p-polarized waves within the framework of the SCIB model, corresponding to a flat interface between a semi-infinite dielectric (occupying the $z < 0$ half-space) and a nonlocal medium, i.e., the superconductor (spanning the $z > 0$ half-space).

To that end—assuming, without loss of generality, that the system is uniform along the $\hat{\mathbf{y}}$—we write the electric field as $\mathbf{E}(\mathbf{r},t) = \mathbf{E}(q_\parallel,\omega;z)\mathrm{e}^{\mathrm{i}(q_\parallel x - \omega t)}$, where $\mathbf{E}(q_\parallel,\omega;z)$ can be expressed as a Fourier integral over $q_\perp$

$$\mathbf{E}(q_\parallel,\omega;z) = \frac{1}{2\pi}\int_{-\infty}^{\infty} \mathbf{E}(q_\parallel, q_\perp,\omega)\mathrm{e}^{\mathrm{i}q_\perp z} dq_\perp, \tag{4}$$

where $q^2 = q_\parallel^2 + q_\perp^2$. The SCIB formalism consists in solving the field equations for the whole space with additional boundary conditions at $z = 0$ that are consistent with specular reflection† at that plane‡ (hence, this approximation neglects quantum

*The interested reader may find more details, for instance, in Refs. (4–7)

†For this reason, the SCIB model is also sometimes referred to as the "specular-reflection model" (SRM) (9).

‡Thereby enforcing the relations: $E_x(z) = E_x(-z)$ and $E_z(z) = -E_z(-z)$.

interference effects arising from the interaction between the incoming and reflected parts of the electron wave function). Then, after some algebra, the components of the electric field inside the superconductor read

$$E_x(q_\perp) = \frac{2g_x}{q^2}\left[\frac{q_\parallel^2}{\frac{\omega^2}{c^2} + \mathrm{i}\mu_0\omega\,\sigma_\mathrm{L}(q,\omega)} + \frac{q_\perp^2}{\frac{\omega^2}{c^2} + \mathrm{i}\mu_0\omega\,\sigma_\mathrm{T}(q,\omega) - q^2}\right], \quad [5]$$

$$E_z(q_\perp) = -\frac{2g_x}{q^2} q_\parallel q_\perp \frac{q^2 + \mathrm{i}\mu_0\omega\left[\sigma_\mathrm{L}(q,\omega) - \sigma_\mathrm{T}(q,\omega)\right]}{\left[\frac{\omega^2}{c^2} + \mathrm{i}\mu_0\omega\,\sigma_\mathrm{L}(q,\omega)\right]\left[\frac{\omega^2}{c^2} + \mathrm{i}\mu_0\omega\,\sigma_\mathrm{T}(q,\omega) - q^2\right]}, \quad [6]$$

with $c^{-2} = \epsilon_0\mu_0$, and where

$$g_x \equiv \left.\frac{dE_x(z)}{dz}\right|_{z=0^+} - \mathrm{i}q_\parallel E_z(z=0^+). \quad [7]$$

We now have to impose Maxwell's boundary conditions (6, 8) on these fields to study their coupling to electromagnetic fields outside the superconductor. In the dielectric half-space, we model the dielectric through a relative permittivity ϵ_d and assume a field inside the dielectric of the form $\mathbf{E}_0(\mathbf{r},t) = \mathbf{E}_0(z)\,\mathrm{e}^{\mathrm{i}(k_\parallel x - \omega t)}$, with

$$\mathbf{E}_0(z) = \begin{bmatrix} E_{0i}^x \\ 0 \\ E_{0i}^z \end{bmatrix} \mathrm{e}^{\mathrm{i}k_{z,\mathrm{d}}z} + \begin{bmatrix} E_{0r}^x \\ 0 \\ E_{0r}^z \end{bmatrix} \mathrm{e}^{-\mathrm{i}k_{z,\mathrm{d}}z}, \quad [8]$$

where $k_{z,\mathrm{d}} = \sqrt{\epsilon_\mathrm{d}\frac{\omega^2}{c^2} - q_\parallel^2}$. We then require that the tangential component of the electric and magnetic fields are continuous across $z = 0$. After some algebra, we obtain the sought-after reflection coefficient for p-polarized waves associated with the dielectric–superconductor interface:

$$r_p^{\text{SCIB}} = \frac{k_{z,\mathrm{d}} - \epsilon_\mathrm{d}\,\Xi}{k_{z,\mathrm{d}} + \epsilon_\mathrm{d}\,\Xi}, \quad [9a]$$

with

$$\Xi \equiv \frac{\mathrm{i}}{\pi}\frac{\omega^2}{c^2}\int_{-\infty}^{\infty} dq_\perp \frac{\mathrm{e}^{\mathrm{i}q_\perp 0^+}}{q^2}\left[\frac{q_\parallel^2}{\frac{\omega^2}{c^2} + \mathrm{i}\mu_0\omega\,\sigma_\mathrm{L}(q,\omega)} + \frac{q_\perp^2}{\frac{\omega^2}{c^2} + \mathrm{i}\mu_0\omega\,\sigma_\mathrm{T}(q,\omega) - q^2}\right]. \quad [9b]$$

To solve this integral above we assume that, for a given frequency ω, the integrand has only two simple poles (3, 7), q_L and q_T, corresponding, respectively, to the zeros of

$$\frac{\omega^2}{c^2} + \mathrm{i}\mu_0\omega\,\sigma_\mathrm{L}(q,\omega) \qquad \text{and} \qquad \frac{\omega^2}{c^2} + \mathrm{i}\mu_0\omega\,\sigma_\mathrm{T}(q,\omega) - q^2. \quad [10]$$

Using the expressions (2) and (3) for the longitudinal and transverse components of the conductivity tensor, we obtain

$$(cq_L)^2 = \frac{\omega^2 - \omega_p^2}{3\bar{\alpha}(\omega,T)} \qquad \text{and} \qquad (cq_T)^2 = \frac{\omega^2 - \omega_p^2}{1 + \bar{\alpha}(\omega,T)\left(\frac{\omega_p}{\omega}\right)^2}, \quad [11]$$

respectively. We then employ the residue theorem to determine Ξ; specifically, the residues read

$$\mathcal{R}_L = -\left[6q_L^\perp\frac{\omega^2}{\omega_p^2}\bar{\alpha}\right]^{-1}, \qquad \text{and} \qquad \mathcal{R}_T = -\left[2\left(1 + \frac{\omega_p^2}{\omega^2}\bar{\alpha}\right)q_T^\perp\right]^{-1}, \quad [12]$$

where $q_\parallel^2 + (q_{T,L}^\perp)^2 = q_{T,L}^2$. Hence, we find

$$\Xi(q_\parallel,\omega) = \frac{\omega^2}{\omega^2 - \omega_p^2}\left[\left(\frac{\omega_p}{\omega}\right)^2\frac{q_\parallel^2}{q_L^\perp} + q_T^\perp\right]. \quad [13]$$

In this derivation we have neglected any relaxation mechanisms for the sake of clarity. The main effect of considering finite loss (like we do in our calculations), in the form of an energy-independent phenomenological relaxation rate γ, is that the function $\bar{\alpha}(\omega,T)$ acquires an energy-dependent imaginary part, with a sharp feature around $2\Delta_0(T)$.

Applicability and limitations of the SCIB model. The general appeal of the SCIB model rests upon its ability to incorporate the salient aspects of nonlocal (i.e., q-dependent) response in a relatively simple fashion (4–6, 9), taking only the material's bulk response as input [here, the components of the conductivity tensor, $\sigma_\mathrm{L}(q,\omega)$ and $\sigma_\mathrm{T}(q,\omega)$]. Nevertheless, the SCIB model admittedly has limitations in describing *surface* effects as it relies on the material *bulk* response. As such, the presence of interfaces or surfaces are accounted for by imposing electromagnetic boundary conditions at the said interface(s).

Further, within the SCIB model, the carriers are assumed to be specularly reflected at the surface thereby neglecting interference between incident and scattered carriers (4, 9). In spite of this, the SCIB model is expected to be qualitatively accurate in the present setting, namely, the incorporation of the superconductor's nonlocal response and ensuing observation of an anticrossing feature in the graphene plasmon's spectrum, and also the description of a sharp feature in the LDOS enhancement centered about the frequency of the superconductor's Higgs amplitude mode. Finally, although one could employ more sophisticated approaches, like the infinite or finite quantum barrier models (9), the potential gains would be only quantitative; in fact, a quantitatively rigorous description of a superconductor's nonlocal electrodynamic response likely requires an appropriate description of the anisotropic, layered configuration of most high-T_c superconductors (e.g., in cuprate high-T_c superconductors) and, eventually, many-body interactions (10).

Example: Surface polaritons in a planar vacuum–superconductor interface. Signatures of excitation modes can be found through inspection of the imaginary part of the reflection amplitude, $\operatorname{Im} r_p$ [here computed in accordance with Eq. (9)]. We use parameters for the superconductor that are typical of a high-T_c material, such as YBCO (11): the normal state electron density $n = 6\ \mathrm{nm}^{-3}$, the transition temperature $T_c = 93$ K and the gap at zero temperature $\Delta_0(0) \approx 14.2$ meV. We also assume the gap depends on temperature as $\Delta_0(T) = \Delta_0(0)\sqrt{1-(T/T_c)^{1/4}}$ for $T < T_c$ and is zero for $T > T_c$. A plot of $\operatorname{Im} r_p$ associated with a vacuum–superconductor interface (Fig. S1a) shows the usual surface plasmon polariton feature, characteristic of a normal metal, together with a very faint featuren at an energy close to $2\Delta_0(T) \approx 28.4$ meV. A zoom into this region (Fig. S1b) reveals an (overdamped) anti-crossing between the plasmon branch and the amplitude (Higgs) mode. For this calculation we adopted a relaxation rate $\hbar\gamma = 1\,\mu\mathrm{eV}$, which is well into the clean limit for real samples of high-T_c superconductors (12). In order to observe a well-defined mode in this region we found it necessary to reduce $\hbar\gamma$ even further to $5\times10^{-2}\,\mu\mathrm{eV}$ (Fig. S1c). We note that both the energy and wave vector scales are very small compared to the characteristic scales of the system.

For any finite relaxation rate γ, the SPP's wave vector will acquire an imaginary part. In Fig. S2a, we show the dispersion relation of SPPs in a superconductor, and in Fig. S2b we show the corresponding imaginary part of the SPP wave vector as a function of the energy. We have considered two values of $\hbar\gamma$: $5\times10^{-2}\,\mu\mathrm{eV}$ (blue curves) and $1\,\mu\mathrm{eV}$ (red curves). Notice that even for a small relaxation rate ($\hbar\gamma = 1\,\mu\mathrm{eV}$) the anticrossing between the Higgs mode and the SPP is significantly damped.

S1.3. Vacuum–uniaxial dielectric–graphene–uniaxial diectric–superconductor heterostructure. We now consider the case of a superconductor next to a graphene sheet sandwiched between two layers of a uniaxial dielectric, such as hBN—see Fig. S3.

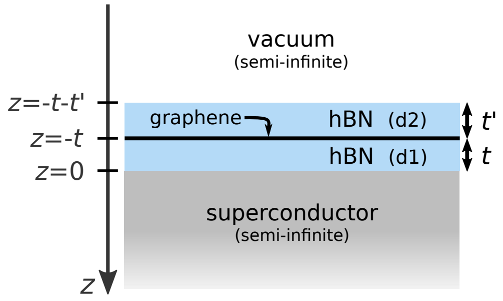

Fig. S3. Schematic of the vacuum–uniaxial dielectric–graphene–uniaxial diectric–superconductor heterostructure under consideration in Sect. S1.3 (here the uniaxial dielectric has been chosen to be hBN for the sake of concreteness).

The graphene sheet is placed at a distance t above the superconductor surface and divide the uniaxial dielectric in two regions: region "d1", between the graphene sheet and the superconductor, with thickness t, and region "d2" with thickness t', between the graphene sheet and vacuum. Our coordinate system (Fig. S3) is defined such that the (semi-infinite) superconductor occupies the half-space $z > 0$. Thus, $z = 0$ corresponds to the interface between the superconductor and region "d1", $z = -t$ corresponds to the position of the graphene sheet and the interface between regions "d1" and "d2", and $z = -t - t'$ corresponds to the interface between region "d2" and the (semi-infinite) vacuum. The dielectric is modeled by the relative permittivity tensor

$$\overleftrightarrow{\boldsymbol{\epsilon}}_{\mathrm{d}} = \begin{pmatrix} \epsilon_{\mathrm{d}}^{x} & 0 & 0 \\ 0 & \epsilon_{\mathrm{d}}^{y} & 0 \\ 0 & 0 & \epsilon_{\mathrm{d}}^{z} \end{pmatrix}. \qquad [14]$$

We assume the electric field in the dielectric to be of the form $\mathbf{E}_{\mathrm{d}l}(\mathbf{r},t) = \mathbf{E}_{\mathrm{d}l}(z)\,\mathrm{e}^{\mathrm{i}(k_\parallel x - \omega t)}$,

$$\mathbf{E}_{\mathrm{d}l}(z) = \begin{bmatrix} E^{x}_{\mathrm{d}l(+)} \\ 0 \\ E^{z}_{\mathrm{d}l(+)} \end{bmatrix} \mathrm{e}^{\mathrm{i}k_{\mathrm{d}}^{\perp} z} + \begin{bmatrix} E^{x}_{\mathrm{d}l(-)} \\ 0 \\ E^{z}_{\mathrm{d}l(-)} \end{bmatrix} \mathrm{e}^{-\mathrm{i}k_{\mathrm{d}}^{\perp} z}, \qquad [15]$$

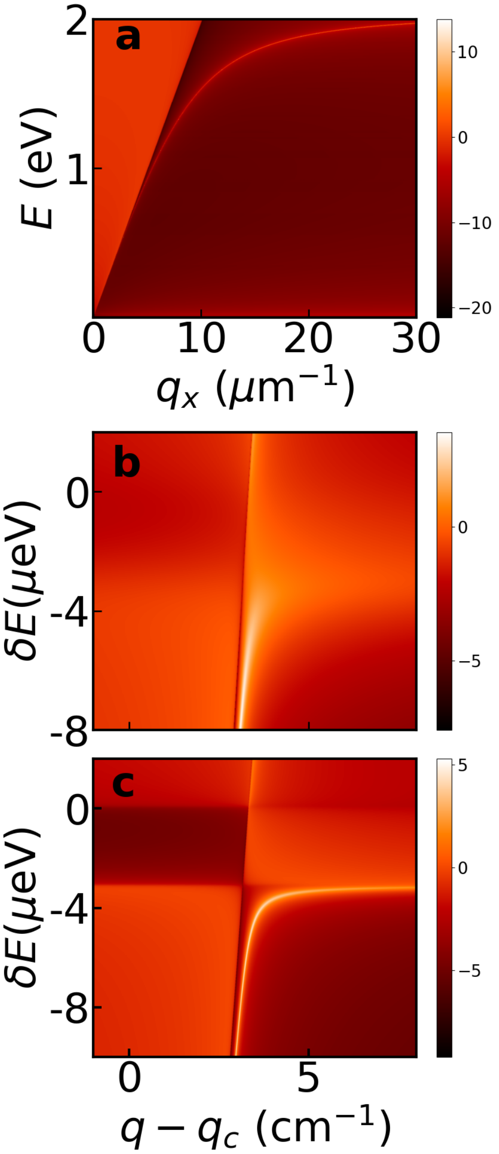

Fig. S1. Logarithm of the imaginary part of the reflection amplitude at the interface between a semi-infinite superconductor and vacuum, at $T = 1$ K and $\hbar\gamma = 1$ µeV. **a,** Energy and parallel wave vector ranges include the usual metallic surface plasmon polariton, seen as a bright line in the plot. **b,** Zoom into a region close to energy of the Higgs mode. Evidence of a crossing between the SPP and the Higgs mode is seen, albeit overdamped. **c,** The same as **b** but with the damping $\hbar\gamma$ drastically reduced to 5×10^{-2} µeV, the anti-crossing between the plasmon and the Higgs mode becomes clear. The dark and light stripes correspond to sign changes of $\log\{\mathrm{Im}\, r_p\}$. These results show that the visibility of the Higgs mode between the interface of superconductor and vacuum can hardly be achieved. In **b** and **c** we defined $\delta E \equiv E - 2\Delta(T)$ and $q_c = 0.1435\,\mu\mathrm{m}^{-1}$.

where $l = 1, 2$ and $k_{\rm d}^{\perp}$ is defined by

$$\frac{\omega^2}{c^2} = \frac{k_{\parallel}^2}{\epsilon_{\rm d}^z} + \frac{(k_{\rm d}^{\perp})^2}{\epsilon_{\rm d}^x}. \qquad [16]$$

We assume an electric field in vacuum of the form $\mathbf{E}_0(\mathbf{r}, t) = \mathbf{E}_0(z)\, \mathrm{e}^{\mathrm{i}(k_{\parallel} x - \omega t)}$,

$$\mathbf{E}_0(z) = \begin{bmatrix} E_{0i}^x \\ 0 \\ E_{0i}^z \end{bmatrix} \mathrm{e}^{\mathrm{i}k_0 z} + \begin{bmatrix} E_{0r}^x \\ 0 \\ E_{0r}^z \end{bmatrix} \mathrm{e}^{-\mathrm{i}k_0 z}. \qquad [17]$$

with $k_0^2 = \frac{\omega^2}{c^2}$. The graphene sheet is modeled by its optical conductivity and appears only in the boundary conditions between media "d1" and "d2". We now require that the tangential components of the electric and magnetic fields are continuous at $z = 0$ and $z = -t - t'$. At $z = -t$, the tangential component of the magnetic field must have a discontinuity due to graphene's

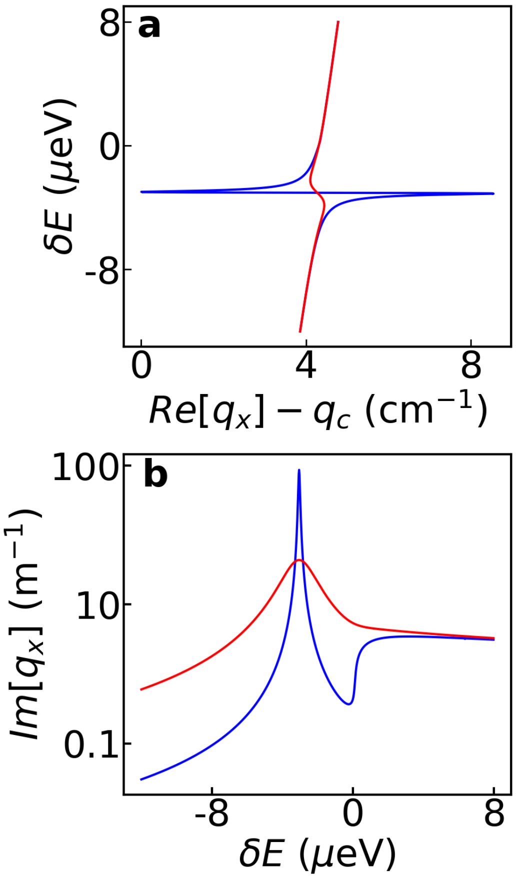

Fig. S2. Dispersion relation (**a**) and imaginary part of the wave vector (**b**) of the superconductor's SPP at the interface with vacuum, at $T = 1$ K. Blue curves correspond to relaxation rate in the superconductor $\hbar\gamma = 5 \times 10^{-2}$ μeV and red curves to $\hbar\gamma = 1$ μeV. The results show that one has to artificially reduce the damping rate in the superconductor for having a sharp, and therefore, easily observable Higgs mode. As we will see, this scenario changes when graphene is located near the superconductor.

finite surface conductivity. The boundary condition at $z = 0$ leads to the following relationship between the magnetic field components in medium "d1",

$$\left(\frac{k_{\rm d}^z}{\epsilon_{\rm d}^x} - k_0^2\Xi\right) H_{\rm d1}^{(+)} = \left(\frac{k_{\rm d}^z}{\epsilon_{\rm d}^x} + k_0^2\Xi\right) H_{\rm d1}^{(-)}. \qquad [18]$$

The boundary condition at $z = -t$ leads to

$$H_{\rm d2}^{(+)} e^{-ik_{\rm d}^\perp t} + H_{\rm d2}^{(-)} e^{ik_{\rm d}^\perp t} - [H_{\rm d1}^{(+)} e^{-ik_{\rm d}^\perp t} + H_{\rm d1}^{(-)} e^{ik_{\rm d}^\perp t}] = \mu_0\omega\sigma_{\rm gr}\Lambda(H_{\rm d1}^{(+)} e^{-ik_{\rm d}^\perp t} - H_{\rm d1}^{(-)} e^{ik_{\rm d}^\perp t}), \qquad [19]$$

$$H_{\rm d1}^{(+)} e^{-ik_{\rm d}^\perp t} - H_{\rm d1}^{(-)} e^{ik_{\rm d}^\perp t} = H_{\rm d2}^{(+)} e^{-ik_{\rm d}^\perp t} - H_{\rm d2}^{(-)} e^{ik_{\rm d}^\perp t}, \qquad [20]$$

where $\sigma_{\rm gr} = \sigma_{\rm gr}(k_\parallel, \omega)$ is graphene's (in general nonlocal) optical conductivity. Finally, the boundary condition at $z = -t - t' \equiv -h$ give

$$H_0^{(i)} e^{-ik_0^\perp h} + H_0^{(r)} e^{ik_0^\perp h} = H_{\rm d2}^{(+)} e^{-ik_{\rm d}^\perp h} + H_{\rm d2}^{(-)} e^{ik_{\rm d}^\perp h} \qquad [21]$$

$$\frac{k_0^\perp}{k_0^2}(H_0^{(i)} e^{-ik_0^\perp h} - H_0^{(r)} e^{ik_0^\perp h}) = \Lambda(H_{\rm d2}^{(+)} e^{-ik_{\rm d}^\perp h} + H_{\rm d2}^{(-)} e^{ik_{\rm d}^\perp h}) \qquad [22]$$

where

$$\Lambda \equiv \frac{k_{\rm d}^\perp \epsilon_{\rm d}^z}{\epsilon_{\rm d}^z (k_{\rm d}^\perp)^2 + \epsilon_{\rm d}^x q_\parallel^2}. \qquad [23]$$

The linear system formed by Eqs. (18)–(22) can be cast in terms of the system's overall reflection amplitude

$$r_p \equiv \frac{H_0^{(r)} e^{ik_0^\perp h}}{H_0^{(i)} e^{-ik_0^\perp h}}, \qquad [24]$$

which is then found numerically.

References

1. Rickayzen G (1965) *Theory of superconductivity.* (Interscience Publishers, New York).
2. Keller O (1990) Electromagnetic surface waves on a cooper-paired superconductor. *J. Opt. Soc. Am. B* 7(11):2229–2235.
3. Keller O, Liu A (1991) Surface polaritons on a BCS-paired jellium. *Opt. Commun.* 80(3):229–232.
4. Ford GW, Weber WH (1984) Electromagnetic interactions of molecules with metal surfaces. *Phys. Rep.* 113(4):195–287.
5. Kliewer KL, Fuchs R (1968) Anomalous skin effect for specular electron scattering and optical experiments at non-normal angles of incidence. *Phys. Rev.* 172(3):607–624.
6. Gonçalves PAD (2020) *Plasmonics and Light–Matter Interactions in Two-Dimensional Materials and in Metal Nanostructures: Classical and Quantum Considerations.* (Springer Nature).
7. Keller O, Pedersen JH (1989) A Nonlocal Description Of The Dispersion Relation And The Energy Flow Associated With Surface Electromagnetic Waves On Metals in *Scattering and Diffraction*, ed. Ferwerda HA. (International Society for Optics and Photonics, SPIE), Vol. 1029, pp. 18–26.
8. Jackson JD (1998) *Classical Electrodynamics.* (Wiley, New York), 3rd edition.
9. Pitarke JM, Silkin VM, Chulkov EV, Echenique PM (2006) Theory of surface plasmons and surface-plasmon polaritons. *Rep. Prog. Phys.* 70(1):1–87.
10. Basov DN, Timusk T (2005) Electrodynamics of high-T_c superconductors. *Rev. Mod. Phys.* 77(2):721–779.
11. (2015) *Physical Properties of High-Temperature Superconductors.* (John Wiley & Sons, Ltd).
12. Orenstein J (2007) *Optical Conductivity and Spatial Inhomogeneity in Cuprate Superconductors*, eds. Brooks J, Schrieffer J. (Springer New York), pp. 299–324.
